# Moran’s *I* quantifies spatio-temporal pattern formation in neural imaging data

**DOI:** 10.1093/bioinformatics/btx351

**Published:** 2017-05-31

**Authors:** Christoph Schmal, Jihwan Myung, Hanspeter Herzel, Grigory Bordyugov

**Affiliations:** 1 Institute for Theoretical Biology, Charité Universitätsmedizin and Humboldt Universität, Berlin, Germany; 2 Wissenschaftskolleg zu Berlin, Berlin, Germany; 3 Computational Neuroscience Unit, Okinawa Institute of Science and Technology, Okinawa, Japan

## Abstract

**Motivation:**

Neural activities of the brain occur through the formation of spatio-temporal patterns. In recent years, macroscopic neural imaging techniques have produced a large body of data on these patterned activities, yet a numerical measure of spatio-temporal coherence has often been reduced to the global order parameter, which does not uncover the degree of spatial correlation. Here, we propose to use the spatial autocorrelation measure Moran’s *I*, which can be applied to capture dynamic signatures of spatial organization. We demonstrate the application of this technique to collective cellular circadian clock activities measured in the small network of the suprachiasmatic nucleus (SCN) in the hypothalamus.

**Results:**

We found that Moran’s *I* is a practical quantitative measure of the degree of spatial coherence in neural imaging data. Initially developed with a geographical context in mind, Moran’s *I* accounts for the spatial organization of any interacting units. Moran’s *I* can be modified in accordance with the characteristic length scale of a neural activity pattern. It allows a quantification of statistical significance levels for the observed patterns. We describe the technique applied to synthetic datasets and various experimental imaging time-series from cultured SCN explants. It is demonstrated that major characteristics of the collective state can be described by Moran’s *I* and the traditional Kuramoto order parameter *R* in a complementary fashion.

**Availability and implementation:**

Python 2.7 code of illustrative examples can be found in the Supplementary Material.

**Supplementary information:**

Supplementary data are available at *Bioinformatics* online.

## 1 Introduction

Oscillatory brain activities emerge with spatial patterns. Historically, purely temporal recordings with very little spatial resolution were used to study processes in the brain. More recently, with improved recording techniques that introduced high spatial resolution, spatial patterning of brain activities began to gain focus. Using measurements from high-density electrodes, a deep sleep electroencephalogram, previously characterized only by the low temporal frequency, is now reinterpreted as a traveling wave (Massimini *et al.*, 2004). Transitions between waking state and coma can be understood as a competition between spatial and temporal instabilities, which causes distinctive spatial patterning (Steyn-Ross *et al.*, 2013). Spatial patterning is also a feature of epilepsy, the pathologically synchronized state once thought to generate a transiently homogeneous state (Muldoon *et al.*, 2013). From the theoretical viewpoint, spontaneous pattern formation in time and space has been understood as a natural consequence of neural network activities (Ermentrout, 1998).

Spontaneous spatial organization is an important universal feature of brain activities, existing both in the normal and in abnormal operating states with unique signature patterns. In this paper, we are interested in a measure of spatial coherence in spatially extended oscillating systems as a consequence of synchronization, in particular on the circadian time scale. As one of the most important characteristics of precise time-keeping, synchronization has usually been assessed quantitatively using the Kuramoto order parameter (Kuramoto, 2012). This order parameter, however, was developed for mean-field coupled systems and does not take into account the spatial organization of the underlying oscillating system. As a consequence, it might sometimes fail to adequately quantify the degree of synchronization in oscillating systems where the spatial extension plays a definitive role.

Our main motivating example here is the circadian spatio-temporal dynamics recorded in the suprachiasmatic nucleus (SCN), which is a networked ensemble of circadian oscillators (Reppert and Weaver, 2002) that can be entrained by external light conditioning (Evans *et al.*, 2013; Myung *et al.*, 2015). A significant portion of SCN neurons has been found to be potent oscillators (Webb *et al.*, 2009). Yet, it is the coupling between them that is thought to be the main contribution to the robustness and precision of the SCN as an oscillator (Aton and Herzog, 2005).

Starting some fifteen years ago, it became possible to look into the spatio-temporal patterns of clock-gene expression in the SCN from living cells by time-lapse microscopy (Kuhlman *et al.*, 2000). A typical technique would be to introduce a reporter construct (either GFP or luciferase) downstream from the promoter region of what is called the ‘clock’ gene (e.g. *Per1*, *Per2*, or *Bmal1*) that is rhythmically expressed or engineered to be fused to a clock protein. In the case of a luciferase reporter system, the strength of bioluminescence is then considered a proxy for the abundance of the product of the target gene (Yoo *et al.*, 2004). Next, the single-cell reporter expression in the intact network of the SCN explant can be observed in real time by a cooled-CCD camera through a microscope, thus providing detailed information on the spatio-temporal pattern formation. In the seminal paper focusing on the synchronization in the SCN (Yamaguchi *et al.*, 2003), it was found that the phase distribution of the rhythmic gene expression across the SCN is governed by the presence of stable patterns. Neurons in different regions of the SCN showed stable phase relations to each other. It was also found that suppressing inter-neuronal communication by applying the sodium channel blocker TTX led to the disruption of the synchronized neuron rhythmicity in the neonatal SCN. Following that paper, a number of technically similar studies appeared, aimed at capturing the features of the spatio-temporal pattern formation of rhythmicity in the SCN, focusing on either the wave-like patterns (Fukuda *et al.*, 2011), regional specificity (Evans *et al.*, 2011), or spatial clustering (Foley *et al.*, 2011; Myung *et al.*, 2012).

To assess the degree of synchronization in such spatio-temporal patterns, the Kuramoto order parameter (Kuramoto, 2012) has been the measure of choice and applied to experimental datasets (Fukuda *et al.*, 2011; Myung *et al.*, 2012). The order parameter accounts for the global degree of the temporal phase coherence among an ensemble of oscillators. In the context of spatio-temporal pattern analysis, the order parameter has a substantial drawback: it does not assume any particular order of interacting oscillators (e.g. the spatial relation of neighbors) and treats all oscillators as indistinguishable from each other. A hypothetical situation with half of the oscillators being out of phase from the other half would result in a zero order parameter. Our intuition, however, tells us that there is a significant amount of order in such a constellation.

Here, we argue that Moran’s *I* (MI) (Moran, 1950) is a more insightful measure for synchronization in oscillators with spatial structure, such as rhythmic gene expression in data series of brain images. The basic idea of MI is to prescribe a notion of distance between each of two oscillating units. If we are interested in patterns in space, the Euclidean distance between units would be the most natural choice.

We start by giving illustrative examples of MI with artificial datasets and explaining typical behaviors of MI depending on the underlying patterns. As a biological application, we turn to the time series of luciferase imaging from the SCN explants. MI reveals spatial order in paradoxical cases, such as the spiral state where Kuramoto order parameter *R* produces a null value. We also show that MI has potential for characterizing the coupling topology of a network. The experimental data reveal cases in which MI and *R* diverge, and it is demonstrated that complementary use of MI and *R* can capture a collective state in fuller detail.

## 2 Materials and methods

### 2.1 Moran’s *I*

‘Spatial autocorrelation’ usually describes the correlation among values of a given variable in dependence on the relative locations between the spatial units (Getis, 2007). If ‘neighbors’ tend to have similar values, one generally speaks of a positive spatial autocorrelation, while one speaks of a negative spatial autocorrelation if they tend to have dissimilar values. One of the most prominent measures of spatial autocorrelation is Moran’s index *I* (MI), which is defined as the ratio between the local and the global coherence in accordance with the following formula:

(1) $$I=\frac{1}{\sum_{ij}w_{ij}}\frac{\sum_{ij}w_{ij}(X_{i}-\bar{X})(X_{j}-\bar{X})}{N^{-1}\sum_{i}{\left(X_{i}-\bar{X}\right)}^{2}}\;.$$

Here, *N* denotes the number of observables or spatial units $X_{i},i=1,2,\ldots ,N$, while $\bar{X}:=\frac{1}{N}\sum_{i}X_{i}$ is the mean of *X_i_*. The notion of ‘local’ as opposed to ‘global’ is prescribed by the spatial weights matrix *w_ij_* between the *i*-th and *j*-th observables.

### 2.2 Spatial weight matrix

The above formula implies that MI depends on the choice of the neighborhood topology, i.e. the spatial weights matrix *w_ij_*, and hence the arrangement of locations *Z_i_* associated with the random variates *X_i_*. A common ordering is the arrangement of random variates on a grid or lattice, e.g. pixel locations in a digital image or two-dimensional cellular automata based on square cells (Moore, 1962). In such a case, the locations $Z_{i}=(x_{i},y_{i})$ are tuples representing coordinates on a two-dimensional raster. A natural definition of adjacency can be then given, e.g. by the von Neumann neighborhood of range *r* via

(2) $$N_{r}(x_{i},y_{i})=\{(x,y):|x-x_{i}|+|y-y_{i}|\leq r\}$$

of a given location (*x_i_*, *y_i_*), leading to the corresponding weight matrix

(3) $$w_{ij}:=\{\begin{array}{c} 1\;\;\text{if}\;\;j\in N_{r}(x_{i},y_{i})\;\;\text{and}\;\;i\neq j\\ 0\;\;\text{in}\;\text{all}\;\text{other}\;\text{cases}\;. \end{array}$$

It is often the case that the locations *Z_i_* of the random variates of interest *X_i_* are not arranged in a regular pattern such as grids, but can be distributed freely on a two-dimensional plane. Under such circumstances, a common definition (Getis, 2007) of a spatial weight matrix is based on the inverse Euclidean distance between two locations *Z_i_* and *Z_j_* and is given by

(4) $$w_{ij}:=\{\begin{array}{c} ||Z_{i}-Z_{j}|{|}_{2}^{-\alpha },\;\;\text{if}\;\;\;i\neq j\\ 0\;,\;\;\text{else}\; \end{array}$$

where *α* denotes the decay parameter specifying the typical interaction range.

### 2.3 Image analysis and time series extraction

Previously published bioluminescence time-lapse recordings are analyzed for their spatio-temporal order in Sections 3.3 and 3.4. In Section 3.3, average intensity values are calculated from square-shaped regions of interest (ROIs) that are arranged as a grid that overlays the original image (Myung *et al.*, 2012). The edge lengths of the ROIs are usually chosen to approximately resemble the size of a single neuron (Myung *et al.*, 2012). In Section 3.4, time-series data and locations of the circular ROIs that correspond to single neurons have been obtained directly from the published dataset (Abel *et al.*, 2016). For both datasets, the corresponding time-series data is baseline-detrended by means of a Hodrick-Prescott filter, as previously described (Myung *et al.*, 2012; St. John and Doyle, 2015).

### 2.4 Instantaneous phase

We extracted the instantaneous phase θ(t) and amplitude *A*(*t*) of a given time series *s*(*t*) by means of the analytic signal z(t)=s(t)+i H(s(t)), where $H(s(t)):=\pi^{-1}\;p.v.\int_{R}d\tau \;\frac{s(\tau )}{t-\tau }$ is the Hilbert transform, with the integral being evaluated in the sense of Cauchy’s principle value. Within this two-dimensional embedding in the complex plane, the instantaneous phase and amplitude can be naturally defined via θ(t):=arg⁡(z(t)) and $A(t):=\sqrt{ℜ{\left(z(t)\right)}^{2}+ℑ{\left(z(t)\right)}^{2}}$, respectively. Here, ℑ(z(t)) and ℜ(z(t)) denote the imaginary and real part of the complex analytic signal *z*(*t*) at time *t*, respectively, and arg⁡(z(t)) is the argument of *z*(*t*). Note that arg⁡(z(t)) can be easily computed by the atan2(ℑ(z(t)), ℜ(z(t))) function that computes the $\arctan\left(\frac{ℑ(z(t))}{ℜ(z(t))}\right)$ function with respect to the signs of ℜ(z(t)) and ℑ(z(t)). Throughout this paper, Hilbert transformation of discrete real-valued experimental circadian time-series is performed numerically by the SCIentificPYthon function hilbert.

### 2.5 Circular statistics and an alternative Moran’s *I*

Since the domain of instantaneous phases θ(t), numerically obtained as described in Section 2.4, is cyclic in the range −π<0≤π, descriptive statistics cannot be attempted without defining an appropriate distance measure. In this cyclic dataset, Euclidean distances can fail to measure the displacement of two adjacent points on the unit circle, e.g. point $x_{2}=\pi$ and $x_{1}=-\pi +\epsilon$ are separated by $x_{2}-x_{1}=2\pi -\epsilon$. A convenient way of defining a distance measure for circular data is to regard each phase as a point on the unit circle of the complex plane. From standard arithmetic with complex numbers, it follows that the distance between two phases *X*_1_ and *X*_2_ on the unit circle $S_{1}$ is given by $d_{\theta }(X_{1},X_{2}):=\text{atan}2(\text{sin}\;(X_{1}-X_{2}),\;\text{cos}\;(X_{1}-X_{2}))$.

Along these lines, the mean value $\bar{X}$ of a given set of phases ${\left\{X_{i}\right\}}_{i=1}^{N}$ can be evaluated via $\bar{X}:=\text{atan}2(\bar{S},\bar{C})$, with $\bar{C}:=\frac{1}{N}\sum_{i=1}^{N}\;\text{cos}\;(X_{i})$ and $\bar{S}:=\frac{1}{N}\sum_{i=1}^{N}\;\text{sin}\;(X_{i})$. Thus, a modified $I_{\theta }$, defined to take into account the cyclic nature of the spatial units *X_i_* of interest, is given by

(5) $$I_{\theta }:=\frac{1}{\sum_{ij}w_{ij}}\frac{\sum_{ij}w_{ij}\;d_{\theta }(X_{i},\bar{X})\;d_{\theta }(X_{j},\bar{X})}{N^{-1}\sum_{i}d_{\theta }{\left(X_{i},\bar{X}\right)}^{2}}.$$

### 2.6 Statistical properties

To assess whether a certain index *I*, calculated for a given set of values *X_i_* and a well-defined weight matrix *w_ij_*, deviates significantly from the null hypothesis of no spatial autocorrelation, we first determine the sampling distribution of *I* under such a null hypothesis. Two conceptually different ways to obtain the sampling distribution have been proposed, depending on how the null hypothesis of no spatial autocorrelation is realized. One can either randomly sample the values of the spatial units *X_i_* from a given distribution (re-sampling approach), or randomly shuffle (randomization approach) the positions of the spatial units for a fixed set of values *X_i_* (Cliff and Ord, 1971; Goodchild, 1986). Throughout this paper, we computationally determine the sampling distribution of *I* values under both realizations of the null hypothesis using the Monte-Carlo approach. Subsequent statistical inferences, i.e. whether an observed value of *I* statistically significantly deviates from the null hypothesis, are based on these Monte-Carlo sampling distributions.

### 2.7 Dynamical systems simulations

Systems of ordinary differential equations as given by Equation (8) are solved using the SCIentificPYthon function odeint.

## 3 Main results

### 3.1 Intuition behind Moran’s *I* and its distribution

**Fig. 1.**
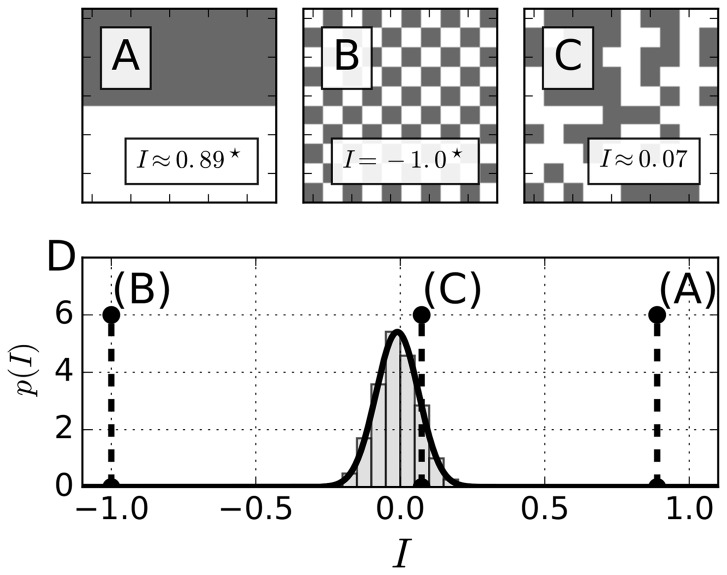
Moran’s *I* detects non-random spatial patterns. (**A–C**) Illustrative examples of spatial distributions on a binary 10 × 10 grid where each grid cell either adopts a value of 0 (white) or 1 (gray). D) Distribution of all possible values of *I* on a 10 × 10 grid conditioned to a von Neumann neighborhood of range *r* = 1. The histogram was obtained by determining *I* for $N=10^{6}$ randomly generated patterns under the assumption of an unbiased probability of occurrence of the binary values. The bold black line denotes a fit of a normal distribution to the data, resulting in μ≈−0.01 and σ≈0.07. Dashed black lines highlight the specific indices *I* determined for the patterns in panels (A)–(C)

To get an intuition of how Moran’s *I* (MI) measures the degree of spatial coherence in two-dimensional patterns, we start with synthetic data and its interpretation in terms of MI in Figure 1. The panels (A), (B) and (C) show three different examples of spatial patterns with qualitatively different values of *I*. In all three cases, the spatial weights *w_ij_* are chosen to be one for immediate neighbors in the same row or column, and otherwise zero, as determined by Equation (3).

First, as illustrated in Figure 1(A), the value of MI is close to one if the majority of the domain is occupied by homogeneous patterns. The high positive value of *I* reflects the fact that the majority of the cells are in the same or a similar state as their neighbors and the number of cells that differ from their neighbors is small. In contrast, Figure 1(B) shows a pattern in which every cell is in a state opposite that of its four immediate neighbors. This dominance of the state opposition is reflected by the negative value of *I* = –1 for the pattern observed in Figure 1(B). Last, in Figure 1(C), there is an intermediate case where values in each cell are picked randomly from zero and one with an unbiased probability of 0.5. Despite some subjective resemblance to a spatial pattern in Figure 1(C), Moran’s *I* recognizes the randomness of the distribution of cell values and produces a value very close to zero. In Figure 1(C), cells on average have an equal number of neighbors in the same as in the opposite state.

MI can also be used to prescribe statistical significance to observed patterns. In Figure 1(D) we plot the histogram and the fitted distribution of *I* for the 10 × 10 grid geometry as in Figure 1(A)–(C), obtained by numerical sampling with binary values randomly prescribed to the elements of the grid. The resulting distribution of *I* can be approximated by a normal distribution with mean μ≈−0.01 and a standard deviation of σ≈0.07. Comparing the values of MI from Figure 1(A)–(C), we see that both (A) (nearly perfect spatial coherence) and (B) (the highest possible degree of spatial incoherence) indeed represent extremely rare events, sitting on the tails of the distribution. The case in Figure 1(C), in contrast, lies close to the mean value of the distribution and is hence characterized by a larger probability to be found in a random experiment.

### 3.2 Using Moran’s *I* to measure synchronization in oscillating lattices

We now turn to making use of MI to characterize dynamic synchronization processes on a lattice of locally coupled oscillatory units. The motivation for using an alternative measure of synchronization in spatially extended oscillating systems is the following. Originally, the complex order parameter

(6) $$R(t)e^{i\psi (t)}:=\frac{1}{N}\sum_{j=1}^{N}e^{i\theta_{j}(t)}$$

was introduced by Kuramoto to characterize the degree of synchronization in globally coupled oscillator systems. Here, R(t)∈[0,1] measures the phase coherence, while ψ(t) denotes the ensemble average over all phases $\theta_{j}(t)$. In this measure, spatial neighborhoodness between oscillators is not present and the presence of local coherence is completely ignored. In the cases of oscillators arranged in a two-dimensional array with local interactions, the global order parameter fails to quantify the degree of synchronization in the emerging pattern.

To make this point clear, we contrast the Kuramoto order parameter and MI in assessing spatial autocorrelation in a two-dimensional oscillating system. The model most widely used to study synchronization phenomena in large populations of oscillating units is the Kuramoto model (Kuramoto, 1975), governed by the equation

(7) $${\dot{\theta }}_{i}(t)=\omega_{i}+\sum_{j=1}^{N}\Gamma_{ij}(\theta_{j}(t)-\theta_{i}(t))\;.$$

Each of the *N* oscillators is described by a single phase variable $\theta_{i}(t)\in S_{1}$ that has an intrinsic frequency *ω_i_* and is influenced by any other oscillator *j* via the coupling function $\Gamma_{ij}(\theta_{j}(t)-\theta_{i}(t))$. In the following, we consider a set of Kuramoto oscillators that couple through nearest neighbor interactions, i.e.

(8) $${\dot{\theta }}_{i}(t)=\omega_{i}+K\sum_{j\in N_{1}(x_{j},y_{j})}\;\text{sin}\;(\theta_{j}(t)-\theta_{i}(t)),$$

a system that has been shown to obey a variety of interesting spatial dynamics, such as the emergence of phase clustering, phase waves, or spirals (Acebrón *et al.*, 2005; Ermentrout and Ko, 2009; Kuramoto, 2012). The control parameters are the coupling strength *K* between the oscillators and the spread of their intrinsic frequencies $\omega_{i}=\frac{2\pi }{\tau_{i}}$. Each of the intrinsic periods $\left(\tau_{i}\right)$ has been sampled from a normal distribution with a mean value of *μ* = 24 h and a standard deviation of σ≈2 h, which approximately resembles the period dispersal of single SCN neurons from mice in the absence of cell-to-cell communication (Bloch *et al.*, 2013; Herzog *et al.*, 2004; Liu *et al.*, 2007).

**Fig. 2.**
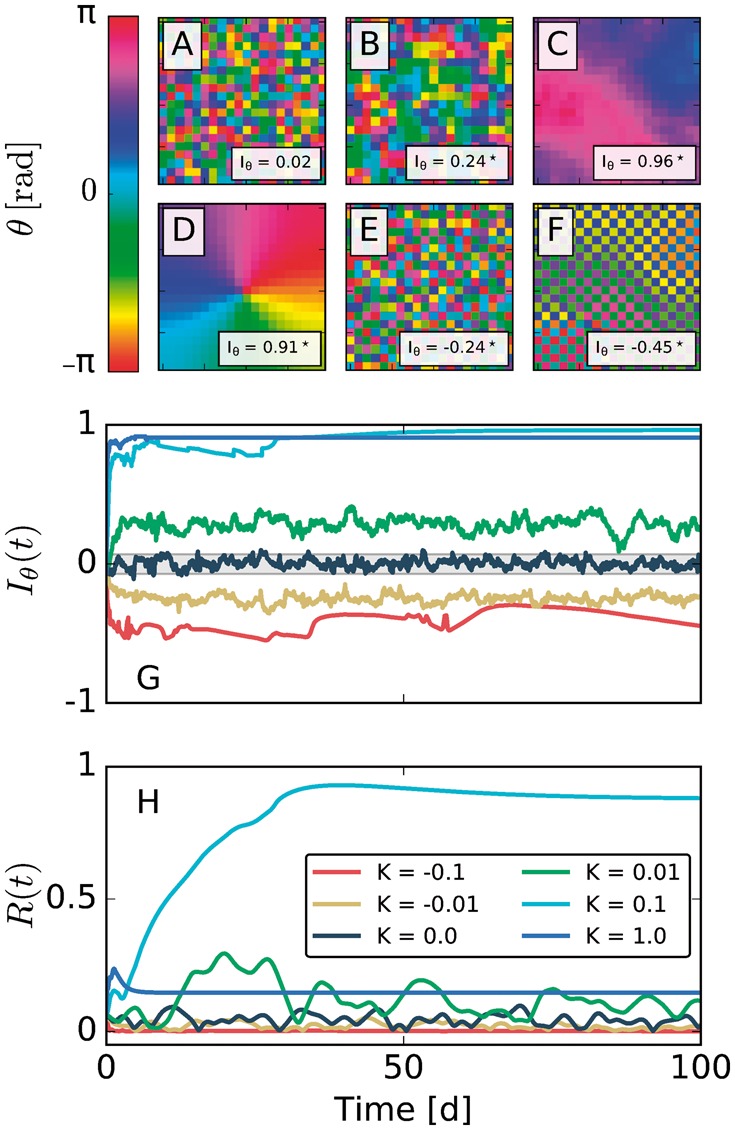
Moran’s $I_{\theta }$ reliably detects spatial ordering in the phase dynamics of a two-dimensional array of coupled Kuramoto phase oscillators with nearest neighbor interactions. (**A–F**) Spatial phase distributions at time t=100 d for different values of coupling strength *K* but identical distributions of initial conditions and intrinsic periods that were sampled from a uniform as well as a normal distribution, respectively. The corresponding coupling strengths *K* in panels (A-F) are K=0,0.01,0.1,1,−0.01,−0.1, respectively. G-H) Dynamical evolution of Moran’s index $I_{\theta }(t)$ and the phase coherence *R*(*t*). Note that $I_{\theta }(t)$ and *R*(*t*) correspond to the same simulations that lead to the phase distributions at t=100 d as represented in panels (A-F). The gray-shaded area depicts the range of $I_{\theta }$ values for which the null hypothesis of no spatial autocorrelation cannot be withdrawn at a significance level 0.05 with a two-sided test. The corresponding sampling distribution is depicted in Supplementary Figure S3

In Figure 2A–F, we plot representative solutions for different values of *K*, captured at the end (*t* = 100 days) of the integration time to remove the transient dynamics following initial conditions. Figure 2G and H show the dynamical evolution of Moran’s index $I_{\theta }(t)$ and the global phase coherence *R*(*t*) for different values of *K*, respectively. Each simulation starts from the same set of randomly generated initial conditions and an identical distribution of intrinsic frequencies. At first glance, we see that increasing the coupling strength *K* between the oscillators in the array leads to increasing synchronization among them and, as a rule, to an increase in $I_{\theta }(t)$: while $I_{\theta }(t)$ mostly fluctuates around a value close to zero in the absence of coupling with *K* = 0 (see Fig. 2A and G), phase clusters that increase in size emerge with an increasing coupling strength of *K* = 0.01 and *K* = 0.1 (compare Fig. 2B and C), respectively. After a short transient dynamics, $I_{\theta }(t)$ evolves toward higher values and the null hypothesis of no spatial autocorrelation can be rejected with a high degree of certainty (*P* < 0.05). A further increase in coupling strength to a value of *K* = 1 leads to the formation of a stable spiral with a center close to the midpoint of the two-dimensional array (see Fig. 2D). Since the phases are arranged nearly radially around the center of the spiral across their whole co-domain, the mean-field phase coherence *R*(*t*) adopts low values, close to those in the case of *K* = 0.01, see Figure 2H. Without prior knowledge of the spatial phase ordering as seen in Figure 2D, this thus does not speak in favor of a high degree of synchrony. In this case, $I_{\theta }(t)$ is much more reliable.

The high probability of a cell having a neighbor with a proximal phase is reflected by high values of $I_{\theta }(t)$, and hence the null hypothesis of no spatial autocorrelation can be rejected with a high degree of certainty (*P* < 0.05, see also Fig. 2G). A similar picture can be drawn in the case of negative, i.e. *K* < 0, nearest-neighbor couplings. In the negative coupling case, two interacting oscillators tend to attract each other when they are anti-phasic and push each other apart when their phases are close. Such a tendency to adopt phases that are in anti-phasis with respect to the nearest neighbors leads to low values of the classical mean-field phase coherence *R*(*t*), suggesting no synchrony of the ensemble (compare Fig. 2E, F and H). Again, the time-dependent form of MI $I_{\theta }(t)$ reliably detects the spatial anti-correlation inherent in the system, while indicating that the assumption of no spatial autocorrelation can be reliably rejected (*P* < 0.05, see Fig. 2G).

**Fig. 3.**
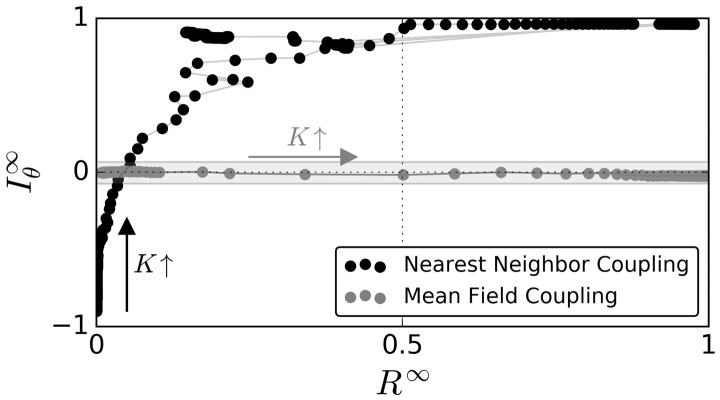
Time averages of steady-state dynamics (i.e. after the decay of transient dynamics) of Moran’s index ($I_{\theta }^{\infty }$) plotted versus the steady state global phase coherence ($R^{\infty }$) for different coupling strength K∈[−0.2,1], either for the case of nearest neighbor couplings (black dots) or a global mean field coupling (gray dots). Details on the numerical calculation of steady state values can be found in the caption of Supplementary Figure S1. Each dot corresponds to one particular simulation for a given coupling strength *K*. Lines connect dots that correspond to experiments sharing the smallest differences in coupling strength *K*. Black and gray arrows denote the direction of increasing coupling strength *K* in case of nearest-neighbor and mean-field couplings, respectively. The gray-shaded area depicts the range of $I_{\theta }$ values for which the null hypothesis of no spatial autocorrelation cannot be withdrawn at a significance level 0.05 with a two-sided test, similar to Figure 2

Figure 3 summarizes these findings by plotting the steady-state values of MI, $I_{\theta }^{\infty }$, against $R^{\infty }$ for different coupling strength *K* either for nearest-neighbor (black dots) or for mean-field (gray dots) coupling. The corresponding Supplementary Figures S1 and S2 plot $I_{\theta }^{\infty }$ and $R^{\infty }$ against different values of *K*. Notice that $I_{\theta }^{\infty }$ and $R^{\infty }$ increase with increasing *K* in the case of nearest-neighbor coupling until the particular dynamical state of a stable spiral emerges. In these cases, which occur at 0.1≲K≲0.124 and K≳2.232 for the particular initial conditions chosen in this example, $R^{\infty }$ drops to lower values while $I_{\theta }^{\infty }$ remains high, indicating a high degree of spatial autocorrelation. The locality of coupling matters, since a mean-field coupling topology leads to qualitatively different results in simulations under the same initial conditions of phases and intrinsic frequencies. In the case of the global coupling, increasing coupling strength generally leads to a higher degree of phase coherence $R^{\infty }$ but no increase in $I_{\theta }^{\infty }$, reflecting the absence of any particular spatial structure in the network of coupled oscillators.

In conclusion, MI is a reliable method for detecting spatial autocorrelation that can be seen as a proxy for synchronization in oscillator ensembles with spatial extension. As we have shown in the synthetic datasets, MI sometimes provides more insights than the classical Kuramoto order parameter *R*(*t*), which fails to recognize synchronization reliably in spatially extended systems with local coupling.

### 3.3 Using Moran’s *I* to measure synchronization in SCN slices

The mammalian master circadian clock SCN has been shown to obey plasticity with respect to experienced environmental cues. In the SCN tissue, re-organizations in spatio-temporal order of clock-gene expression dynamics have been shown to be correlated with the previously applied light schedule. Entrainment of mice to long photoperiods leads to an increasing phase gap of clock-gene oscillations between the ventro-lateral, and dorso-medial neurons in coronal SCN slices when compared with equinoctial (12:12 light:dark) photoperiods (Evans *et al.*, 2013; Myung *et al.*, 2012). Additionally, *in vivo* electrophysiological recordings in the SCN of freely moving mice show broader neuronal activity profiles under long-day conditions than under short-day entrainment (VanderLeest *et al.*, 2007).

To investigate such spatio-temporal re-orderings on different light-schedules, we apply MI to a previously published dataset, where the gene expression of the core clock-gene *Period 2* (Per2) was measured in SCN tissue slices for mice that experienced different photoperiods (Evans *et al.*, 2013). Therein, genetically modified adult male mice, carrying a PER2::LUC reporter construct, were entrained for 12 weeks either under equinoctial or long-day LD20:04 conditions before recording.

**Fig. 4.**
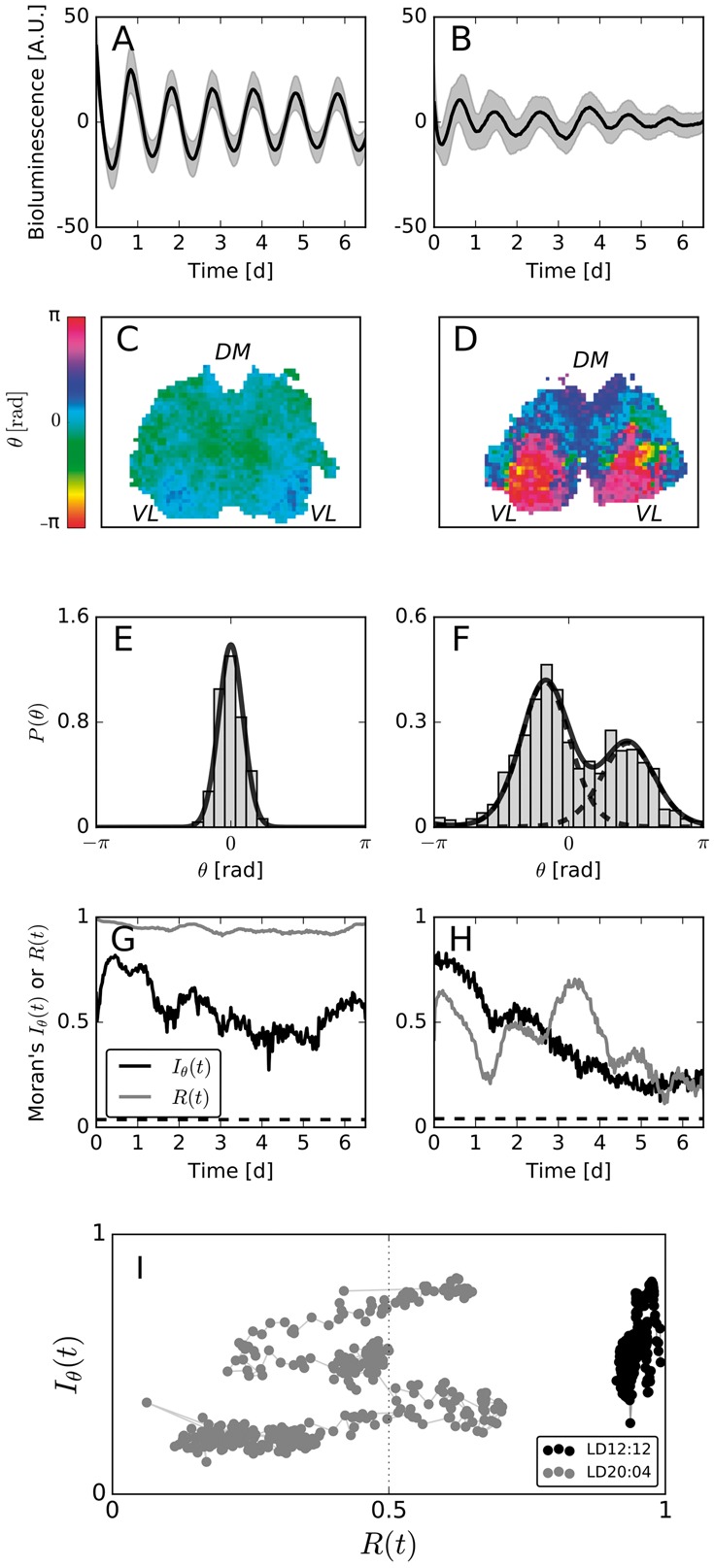
(**A, B**) PER2::LUC expression, averaged over all grid elements that contain SCN tissue (bold black lines), and the corresponding standard deviations (gray shaded area) for each SCN tissue after equinoctial (A) or long-day (B) entrainment. (**C, D**) Color-coded instantaneous phases ${\left\{\theta_{t}\right\}}_{i}$ at time t=20 h of the equinox (C) or long-day (D) entrained tissue. The abbreviations DM and VL indicate rough positions of the dorso-medial and ventro-lateral regions of the SCN, respectively. (**E, F**) Zero-centered histogram of the phase values as depicted in (C) and (D), respectively. (**G, H**) Dynamical evolution of *R*(*t*) (gray bold line) as well as $I_{\theta }(t)$ (black bold line) in case of equinox (G) and long-day (H) entrainment. Dashed black lines correspond to the upper critical values of *I*, estimated computationally from the sampling distribution as described in Section 2.6 at a significance level of 0.05 using a two-sided test. Corresponding sampling distributions are depicted in Supplementary Figure S4. (**I**) Dynamical evolution of Moran’s index and the global phase coherence in the $I_{\theta }(t)-R(t)$ plane. Bold lines connect two circles of $\left(R(t),I_{\theta }(t)\right)$ tuples subsequent to each other

Coronal slices of SCN tissue explants were maintained *ex vivo*, and bioluminescence recordings were made for more than six days using a charge-coupled device (CCD) at a sampling interval of Δt=0.5h, see Figure 4 A and B for the corresponding baseline-detrended time series and (Evans *et al.*, 2011, 2013) for experimental details. After coarse graining the individual images as described in Section 2.3, instantaneous phases $\theta_{i}(t)$ of each grid element *i* are determined by means of a discrete Hilbert transformation as described in Section 2.4. Figure 4 C and D shows two examples of the resulting instantaneous phases ${\left\{\theta_{i}(t)\right\}}_{i}$ estimated at time *t* = 20 hours in the SCN explants from mice entrained under equinoctial or long-day conditions, respectively. The corresponding histograms of the phase values (see Fig. 4E and F) clearly display the emergence of two disjoint phase clusters upon entrainment to long photoperiods, as previously described (Evans *et al.*, 2013). The two clusters correspond to the ventro-lateral (VL) and the dorso-medial (DM) regions of the SCN. Here, the oscillations of the VL region peak earlier than those of the DM region as found previously (Evans *et al.*, 2013). A fit of a bimodal von Mises distribution to the data in Figure 4F reveals a separation of the two clusters’ mean values by Δμ≈1.93rad≈0.61π. Such a strong phase separation leads to a low mean-field phase coherence value of R(t=20h)≈0.49, which contrasts with the high phase coherence observed in the SCN tissue after entrainment to equinox photoperiods (see Fig. 4C and E). Although the global phase coherence differs qualitatively between the two cases, an investigation of Moran’s index $I_{\theta }(t)$ shows that a high degree of spatial autocorrelation is retained for both entrainment conditions. A comparison of the dynamical evolution of *R*(*t*) and $I_{\theta }(t)$ throughout the experiment, as depicted in Figure 4G and H, confirms the findings of the snapshot at t=20 h. While the global phase coherence *R*(*t*) is generally high throughout the *in vitro* recordings in the case of equinoctial entrainment (Fig. 4G), it wobbles around 0.5 in the case of long-day entrainment (Fig. 4H). Although $I_{\theta }$ evolves toward lower values throughout the course of the experiment, the observed patterns significantly deviate from the null hypothesis of no spatial autocorrelation (P≪0.05) at all times *t*.

In conclusion, we state once again that a significant spatial autocorrelation, rather than a high value of the mean field phase coherence *R*(*t*), is indicative of a successful synchronization between SCN neurons. By means of a simultaneous analysis of the dynamical evolution of MI and the global phase coherence in a $I_{\theta }(t)-R(t)$ diagram, as depicted in Figure 4I, one can readily distinguish between qualitatively different synchronization behaviors.

### 3.4 Using Moran’s *I* to measure synchronization among single SCN neurons

In the mammalian circadian clock, inter-cellular coupling between individual SCN neurons has been shown to constitute the quintessential mechanism in generating its remarkable precision, as can be observed on the SCN tissue or behavioral level. This coupling relies on multiple mechanisms, including neuropeptidergical (Aton *et al.*, 2005), gap junction (Shinohara *et al.*, 2000) and synaptic couplings (Yamaguchi *et al.*, 2003). It has been shown, for example, that a pharmacological suspension of synaptic couplings by the application of the action potential blocker *tetrodotoxin* (TTX) can lead to a lower degree of phase coherence and a reduced amplitude of oscillations in clock-gene expression activity in neonatal mice (Yamaguchi *et al.*, 2003).

**Fig. 5.**
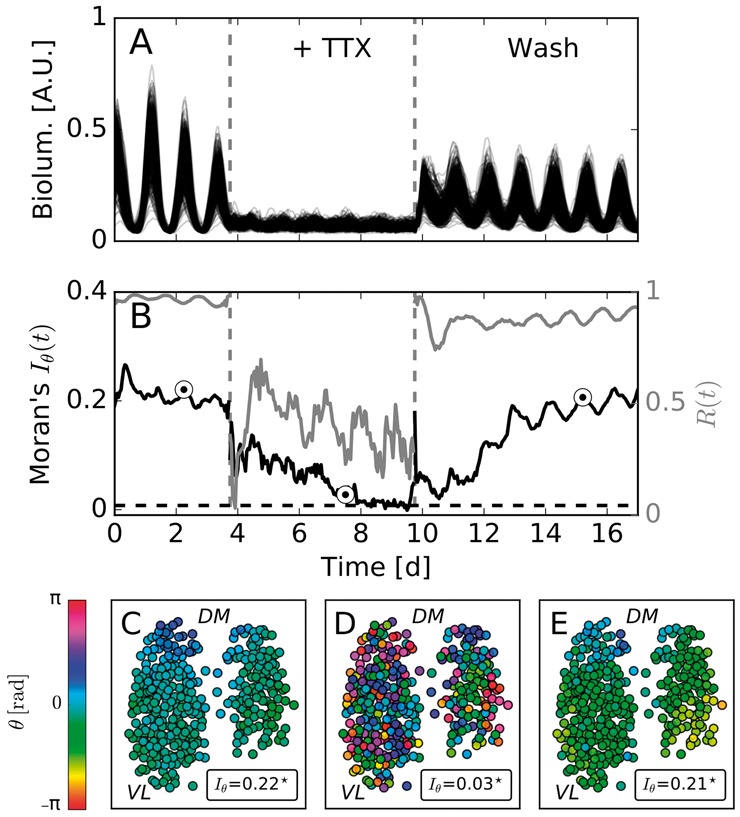
Suspension of synaptic couplings decreases the degree of spatial order in clock gene expression dynamics: (**A**) Bioluminescence time series. (**B**) Moran’s index $I_{\theta }(t)$ (bold black line) and mean-field phase coherence *R*(*t*) (bold gray line) for the time series plotted in the panel (A). (**C–E**) Color coded instantaneous phases $\theta_{i}(t)$ estimated by means of a Hilbert transform. Plotted phase distributions correspond to the time instances depicted by circles in the panel (B). Stars denote statistically significant deviations from the null hypothesis of no spatial autocorrelation. The abbreviations DM and VL indicate rough positions of the dorso-medial and ventro-lateral regions of the SCN, respectively. The dashed black line in (B) corresponds to the upper critical value of $I_{\theta }$, as described in Figure 4. The corresponding sampling distribution is depicted in Supplementary Figure S5

MI can be used to show the effect of TTX on spatial autocorrelation in the SCN-wide circadian oscillations. For this purpose, we re-analyzed a previously published dataset of PER2::LUC bioluminescence recordings in coronal slices of SCN tissue from neonatal to seven-day-old mice that, before being sacrificed, were entrained to equinoctial light-dark cycles. After four days of recordings under culture conditions, TTX was applied to the SCN tissue for a duration of six subsequent days. It was then washed out and the recordings continued for another eight days. By manually identifying and tracking the individual neurons, as done in (Abel *et al.*, 2016), each time trace in the dataset can be associated with the rhythm of a single cell *i* that has a well defined location *Z_i_*. The single cell time traces of every tracked cell under all three conditions are depicted in Figure 5A. Instantaneous phases of these time traces were calculated as described in Section 2.4 after detrending the data by means of a Hodrick-Prescott filter, as described in Section 2.3. Subsequently, $I_{\theta }(t)$ is calculated by using a spatial weight matrix that is based on the inverse Euclidean distance between two locations as given by Equation (4) for *α* = 1. Figure 5B depicts the dynamical evolution of $I_{\theta }(t)$ and *R*(*t*) throughout the experiment. Figure 5C–E shows representative situations from all three conditions.

Initially, all cells show robust coherent circadian rhythms of bioluminescence reporter oscillations (see Fig. 5A). An inspection of the spatial organization of instantaneous phases reveals a phase wave that propagates from the dorsal to the ventral part of the SCN, see Figure 5C for an example. This spatial organization is reflected in a high MI value, i.e. $I_{\theta }\approx 0.22$, which deviates significantly from the null hypothesis of no spatial autocorrelation (P≪0.05). Application of TTX leads to a reduced oscillation amplitude (see Fig. 5A), decreased phase coherence *R*(*t*) as shown in (Taylor *et al.*, 2016) as well as Figure 5B and, on top of that, a gradual decline in spatial autocorrelation as measured by Moran’s index $I_{\theta }(t)$, see Figure 5B and D. Although still significantly different from a purely random pattern (compare Fig. 5B, D and Supplementary Fig. S5), i.e. the probability that adjacent cells have a similar phase is high, $I_{\theta }(t)$ experiences a substantial decrease under TTX. After washing out the neurotoxin, the spatial organization in the form of a phase wave gradually re-emerges while the phase coherence returns to the control level immediately, and Moran’s index $I_{\theta }(t)$ increases until it reaches a value that is comparable to the control condition before the application of TTX.

## 4 Conclusion

The concept of spatial autocorrelation found broad application in diverse disciplines such as geography (Cliff and Ord, 1981; Getis, 2007), ecology (Sokal and Oden, 1978a,b) and economics. Curiously, it escaped the attention of neuroscientists, with a few exceptions, e.g. (Iannella *et al.*, 2010; Lee, 2015; Robinson *et al.*, 2016). One reason for this neglect might be that the spatio-temporal patterns have been difficult to measure from the brain or neural tissues. Another reason might be that a spatio-temporal pattern has not been a clear parameter associated with a neural function and has therefore been discarded as an epiphenomenon.

We showed that a suitable application of MI successfully characterizes spatio-temporal patterning in the collective circadian timekeeping of the SCN neurons. Spatial patterning is one aspect of synchronization in collective activities of neurons that occurs spontaneously. In the modular structure of the brain, this can mean localized activation of specific functional modules. The emergent pattern formation is also a key feature of the spatial sectioning of gene expression during development (Tsiairis and Aulehla., 2016). Adaptation and learning drive complex yet patterned organization, such as pinwheel-like arrangements of orientation columns (Kang *et al.*, 2003). All of these are hallmarks of the synchronized brain state that creates spatially heterogeneous patterns.

Here, we showed that a suitable application of Moran’s *I* (MI), a global index for spatial autocorrelation, can successfully characterize the spatio-temporal patterning of brain activities. The self-contained network of the SCN makes an ideal platform for such studies. It has been shown that by controlling the coupling between neurons in either phase-attractive (positive) or phase-repulsive (negative) polarity, the pattern of oscillation phases in clock-gene expression can be made tunable (Azzi *et al.*, 2017; Myung *et al.*, 2015). Conversely, by observing the spatial patterns, the underlying coupling structure in the network can be deduced, as shown in Section 3.2 for synthetic data, produced by nearest-neighbor coupling versus mean-field coupling in an ensemble of phase oscillators. A proper quantification of patterns is instrumental for this purpose. MI in combination with Monte-Carlo simulations of the underlying sampling distributions flexibly offers a statistical test for the presence of spatial autocorrelation (i.e. patterns) for a broad range of different system sizes, neighborhood specifications (i.e. weight matrices *w_ij_*), and variates (*X_i_*). We introduced a modified index $I_{\theta }$ to identify spatial autocorrelations in systems with cyclic variates such as oscillation phases. On top of that, by plotting the order parameter *R* and *I* in the same plane, we can discriminate qualitatively different synchronization behaviors in both synthetic and experimental data, as we have shown in Sections 3.2–3.4.

Throughout this paper, we applied MI exclusively to spatially extended data in two dimensions. The method can also be adapted to systems in other spatial dimensions. It has the potential to quantify the spatio-temporal organization in a variety of biological and neural systems, such as one-dimensional chains of oscillators (Ermentrout and Kopell, 1984) in developing animals (Horikawa *et al.*, 2006) or three-dimensional fMRI data (Cohen *et al.*, 2017). Finally, it should be noted that any measure of spatial autocorrelation for a given pattern in space depends on the characteristic length one implicitly chooses by defining a specific neighborhood relationship, i.e. the spatial weights *w_ij_*. Spatial correlograms, i.e. plots where *I* is plotted against different distance classes or spatial lags, can be useful for identifying the characteristic length scale of spatially recurrent patterns such as zebra stripes or segmentation patterns that occur in the larval development of insects. There are potential alternatives to MI, such as the phase coherence *R_i_* on spatial subsets *i*. However, its conceptual simplicity and historical use in other fields makes MI an intuitive yet quantitative measure of spatial order in neural imaging data.

## Supplementary Material

Supplementary Data

Supplementary Data
