# Supplementary material for: Moran’s I quantifies spatio-temporal pattern formation in neural imaging data
Source: Bioinformatics. 2017 May 31;33(19):3072–9. doi: 10.1093/bioinformatics/btx351 (PMC5870747; doi:10.1093/bioinformatics/btx351)
Supplement: Supplementary Data [file supportinginformation_btx351.pdf]

Supporting Information

Moran's I quantifies spatio-temporal pattern formation in neural imaging data

Christoph Schmal[1] Jihwan Myung[1 2 3] Hanspeter Herzel[1] Grigory Bordyugov[1]

Supplementary Figures

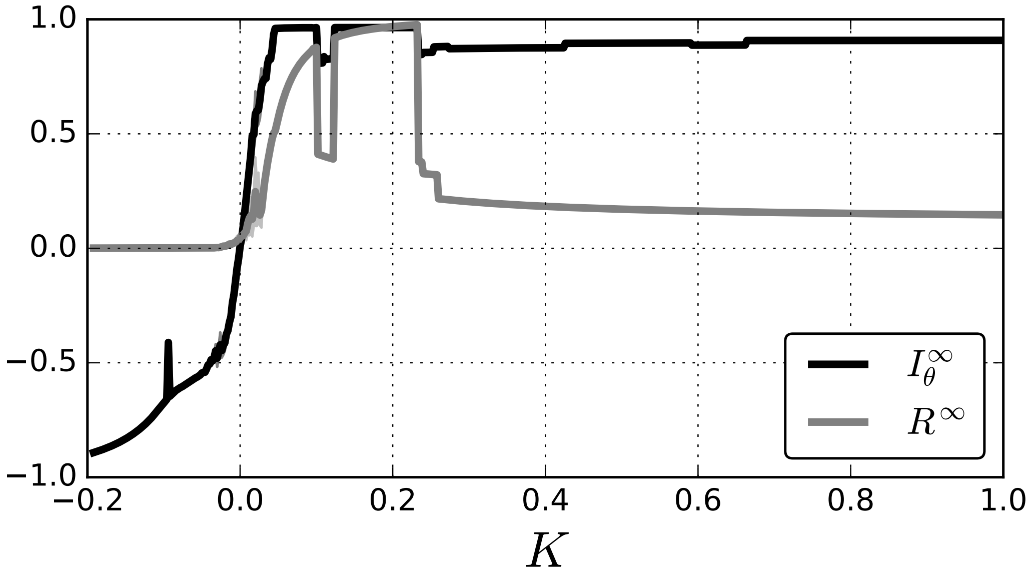

Figure S1: Dependence of the steady-state phase coherence R^∞ and the steady-state values of Moran's index I_θ^∞ on the coupling strength K in the case of *nearest neighbor couplings*. Two abrupt declines of R^∞ due to a formation of a spiral can be observed. The first parameter regime that leads to a formation of a stable spiral can be observed for values that lie approximately between $0.1 \lesssim K \lesssim 0.124$ while the second regime can be observed for $K \gtrsim 2.232$. Note that a minor drop-down of I_θ^∞ can also be observed in these regimes. Steady state values R^∞ and I_θ^∞ have been obtained by time-averaging over the last 2400 h of the simulated values, which is a hundred times of the mean oscillators period in the absence of coupling. The total integration time was $300 \times 24\,\mathrm{h}$. Standard deviations with respect to the time averages have been calculated accordingly and are plotted, though barely visible, as shaded areas around the curves of R^∞ and I_θ^∞. Data represented in this plot is the same as in Figure 3 (black dots).

[1]Institute for Theoretical Biology, Charité Universitätsmedizin and Humboldt Universität, Berlin, D-10115, Germany.
[2]Wissenschaftskolleg zu Berlin, Berlin, D-14193, Germany.
[3]Computational Neuroscience Unit, Okinawa Institute of Science and Technology, Okinawa 904-0495, Japan.

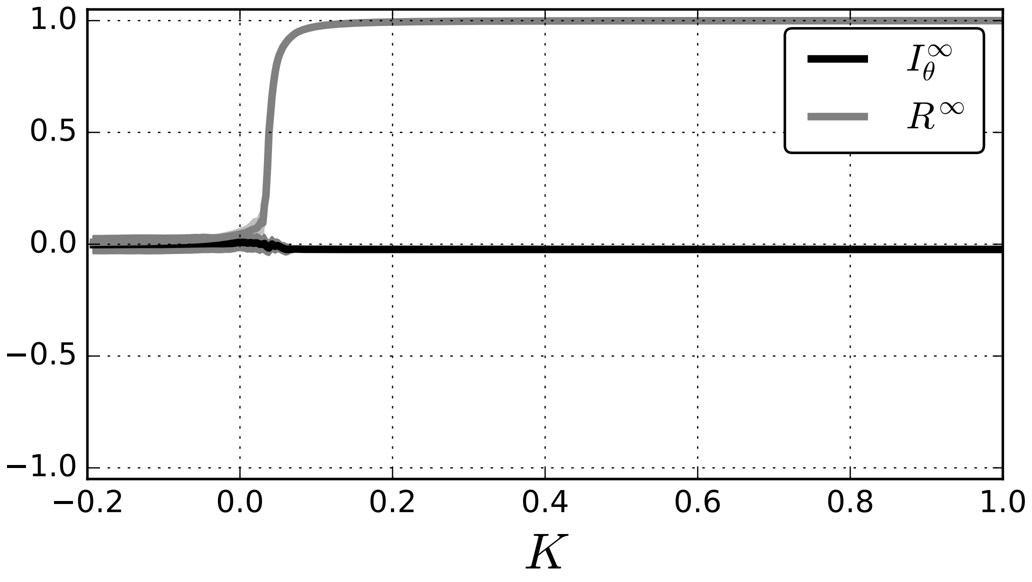

Figure S2: Dependence of the steady-state phase coherence R^∞ and the steady-state values of Moran's index I_θ^∞ on the coupling strength K in the case of *mean field coupling*. Steady state values R^∞ and I_θ^∞ have been obtained by time-averaging over the last 2400 h of the simulated values, which is a hundred times of the mean oscillators period in the absence of coupling. The total integration time was $300 \times 24\,\mathrm{h}$. Standard deviations with respect to the time averages have been computed accordingly and are plotted, though barely visible, as shaded areas around the curves of R^∞ and I_θ^∞. Data represented in this plot is the same as in Figure 3 (gray dots).

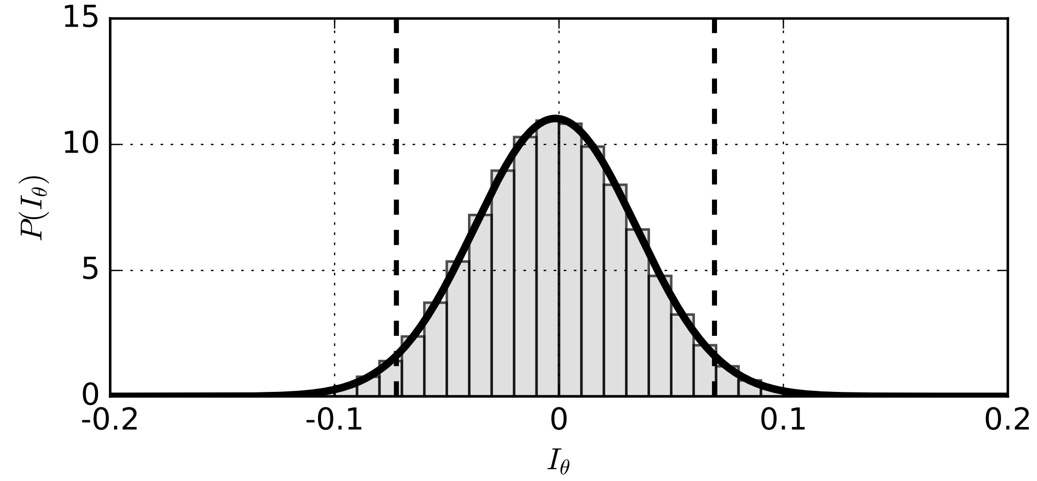

Figure S3: Sampling distribution of Moran's I_θ, using circular statistics under the null hypothesis of no spatial autocorrelation for a 20×20 grid and a weight matrix that considers for a given spatial unit its nearest von Neumann neighbors as investigated in Section 3.2. Monte-Carlo simulations were done as described in Section 2.6 using the re-sampling approach. Sampling distributions, obtained by means of the randomization approach closely match the results from the re-sampling approach (data-not shown). Histograms visualize the data generated via Monte-Carlo simulations. A Gaussian fit to the data, depicted by a bold black line, describes the Monte-Carlo sampling distribution well. Vertical dashed black lines correspond to the critical values of I_θ at a significance level of 0.05 in a two-sided test. The null hypothesis of no spatial autocorrelation cannot be withdrawn for values of I_θ that lie between the upper and these lower critical values. Values of I_θ that are smaller or higher than the lower and upper critical value, respectively, are considered to deviate statistically significantly from the null hypothesis of no spatial autocorrelation under the chosen significance level of 0.05. The range of I_θ values between the lower and upper critical values is depicted by a gray-shaded area in Figure 2 G and Figure 3 of the main text.

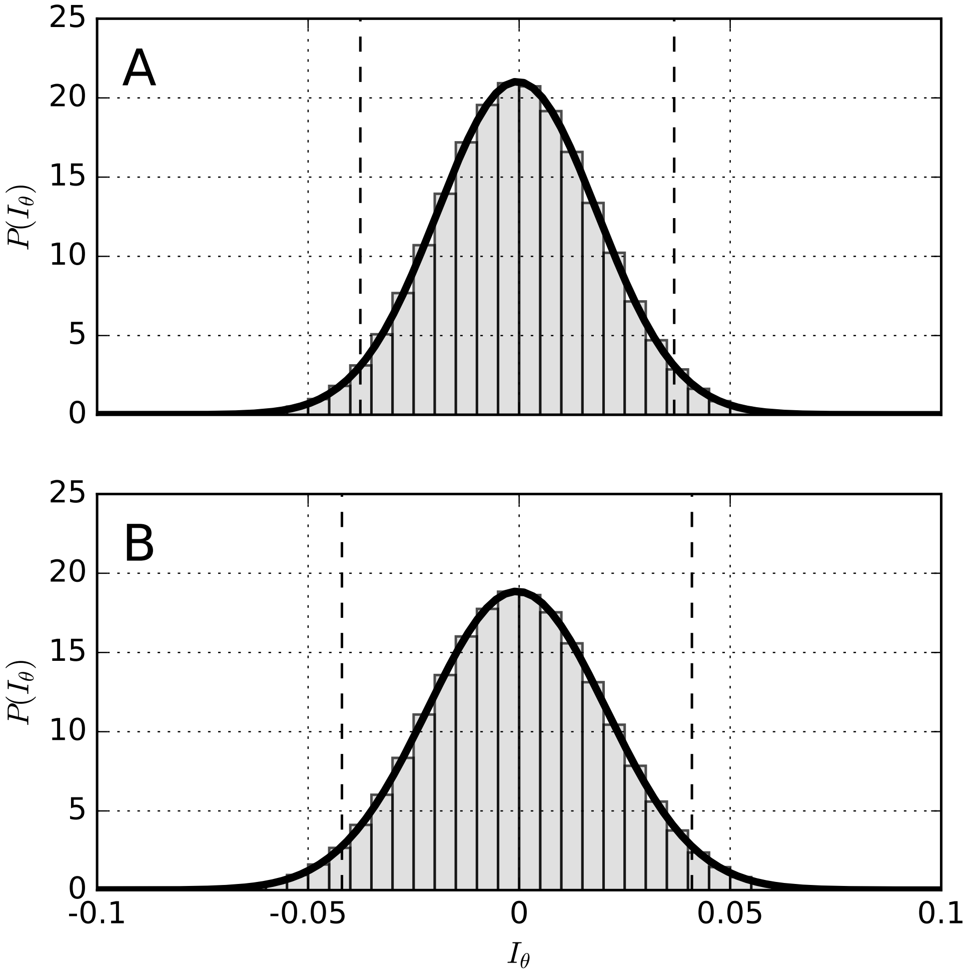

Figure S4: Sampling distribution of I_θ under the null hypothesis of no spatial autocorrelation, using weight matrices as described in Section 3.3. Monte-Carlo simulations were done as described in the caption of Figure S3. While A depicts the sampling distribution obtained with the weight matrix used for equinoctial entrainment data, B depicts the sampling distribution obtained for the long-day entrainment data. Again, a Gaussian distribution (bold black lines) yields a good description of the sampling distribution. Lower and upper critical values of I_θ are depicted by vertical dashed black lines in panels (A)–(B) of this Figure, and by horizontal dashed black lines in panels (G)–(H) of Figure 4 in the main text.

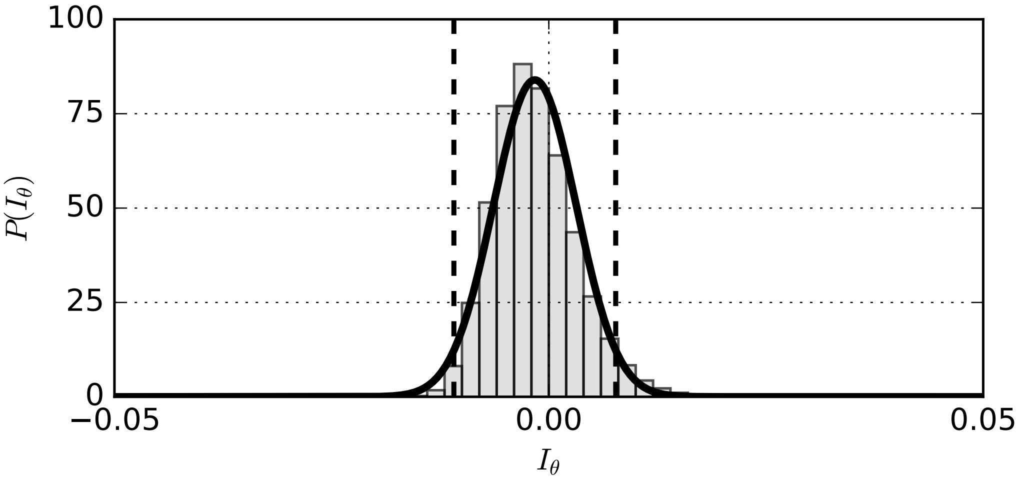

Figure S5: Sampling distribution of I_θ under the null hypothesis of no spatial autocorrelation, using a weight matrices as described in Section 3.4. Although the sampling distribution of I_θ is slightly skewed, a Gaussian distribution (bold black line) still yields a good approximation. The fitted Gaussian slightly over-estimates the mean of the sampling distribution and, thus, slightly underestimates the lower and upper critical values of I_θ. I_θ will, apart from few exceptions, deviate from the null hypothesis of no spatial autocorrelation even after a correction of the underestimated upper critical value. Lower and upper critical values of I_θ are depicted by vertical dashed black lines in this Figure and by horizontal dashed black lines in Figure 5 B of the main text.
